# Leisure-time physical activity and risk of incident cardiovascular disease in Chinese retired adults

**DOI:** 10.1038/s41598-021-03475-6

**Published:** 2021-12-17

**Authors:** Xuanwen Mu, Kuai Yu, Pinpin Long, Rundong Niu, Wending Li, Huiting Chen, Hui Gao, Xingxing Li, Yu Yuan, Handong Yang, Xiaomin Zhang, Mei-an He, Gang Liu, Huan Guo, Tangchun Wu

**Affiliations:** 1 Department of Occupational and Environmental Health, Key Laboratory of Environment and Health, Ministry of Education and State Key Laboratory of Environmental Health (Incubating) School of Public Health, Tongji Medical College, Huazhong University of Science and Technology, 13 Hongkong Rd, Wuhan, 430030 Hubei China; 2 Department of Cardiovascular Diseases, Sinopharm Dongfeng General Hospital, Hubei University of Medicine, Shiyan, 442000 China; 3 Department of Nutrition and Food Hygiene, School of Public Health, Tongji Medical College, Huazhong University of Science and Technology, Wuhan, 430030 China

**Keywords:** Cardiology, Risk factors, Disease prevention, Epidemiology

## Abstract

The optimum amounts and types of leisure-time physical activity (LTPA) for cardiovascular disease (CVD) prevention among Chinese retired adults are unclear. The prospective study enrolled 26,584 participants (mean age [SD]: 63.3 [8.4]) without baseline disease from the Dongfeng-Tongji cohort in 2013. Cox-proportional hazard models were used to estimate hazard ratios (HRs) and 95% confidence intervals (CIs). During a mean 5.0 (1.5) years of follow-up, 5704 incident CVD cases were documented. Compared with less than 7.5 metabolic equivalent of task-hours per week (MET-hours/week) of LTPA, participating LTPA for 22.5–37.5 MET-hours/week, which was equivalent to 3 to 5 times the world health organization (WHO) recommended minimum, was associated with a 18% (95% CI 9 to 25%) lower CVD risk; however, no significant additional benefit was gained when exceeding 37.5 MET-hours/week. Each log10 increment of MET-hours/week in square dancing and cycling was associated with 11% (95% CI 2 to 20%) and 32% (95% CI 21 to 41%), respectively, lower risk of incident CVD. In Chinese retired adults, higher LTPA levels were associated with lower CVD risk, with a benefit threshold at 3 to 5 times the recommended physical activity minimum. Encouraging participation in square dancing and cycling might gain favourable cardiovascular benefits.

## Introduction

Physical inactivity is one of the major modifiable risk factors for cardiovascular disease (CVD)^1,2^, the leading cause of morbidity and mortality globally^3,4^. The World Health Organization (WHO) recommends every adult to maintain a minimum of 150 min of moderate-intensity or 75 min of vigorous-intensity activity per week, which is equivalent to 7.5 weekly metabolic equivalent of task-hours ([MET]-hours/week) of physical activity (PA), to experience health benefits^5^. However, almost one-fourth of the adults worldwide did not meet such recommendations^6^, and even more failed to achieve these levels in post-retirement population^7,8^. In addition to the declining physical function with advancing age^9,10^, pattern and amount of PA in retired adults differ greatly from that in their working counterparts, mainly characterized by a marked reduction in occupational and transportation domains of PA and higher participation in leisure-time physical activity (LTPA)^7,11^. Thus, identifying the optimal amount and type of LTPA for retired adults has become a key priority to guide future recommendation and optimize CVD prevention.

Cumulative studies from western countries have suggested that recreational activities might be more effective to offset the risks of CVD in older adults^12–14^. However, the prospective evidence remained limited regarding the association between incident CVD and recreational activities with Chinese characteristics, such as square dancing, a popular activity enjoyed widespread popularity among middle-aged and older Chinese^15,16^. Furthermore, the prolonged sedentary time in retired adults during the past decades is increasingly striking, which also contributes to an elevated risk of CVD^17–19^. Recent prospective study reported that more activity might attenuate the detrimental association of prolonged sedentary time with CVD^20^. However, most of the previous studies assessed merely common sedentary behaviour such as watching TV^21,22^; it remains unclear about the relation between Mahjong and health status among Chinese^23^.

In the present study of retired adults from the Dongfeng-Tongji cohort, we examined the association of LTPA with the risk of incident CVD and its major components. We also evaluated the dose–response associations of CVD with total and different types of LTPA separately. Additionally, we investigated the association of combined categories of sedentary behaviour and LTPA with the risk of CVD.

## Results

### Baseline characteristics of study participants by LTPA levels

Of the 26,584 participants (43% male and 57% female; mean [SD] age: 63.3 [8.4] years) included in the present study, the median LTPA was 21 MET-hours/week (interquartile range, 11.3–42 MET-hours/week), and 18.2% of the participants (n = 4832) did not meet the WHO recommended PA minimum (< 7.5 MET-hours/week) at baseline. In general, the most common type of leisure activity performed by retired adults was walking (77.7%), followed by square dancing (10.8%), jogging (5.0%) and cycling (4.7%). Table 1 showed the baseline characteristics of study participants according to LTPA categories. Comparing with inactive participants, those with higher levels of LTPA were tended to be educated more, smoke less, drink more, eat more fruit and vegetables, have lower BMI, and have less sedentary time (*P* < 0.01).

**Table 1:** Baseline characteristics of study participants according to leisure-time physical activity.

Characteristics	Leisure-time physical activity, MET-hours/week^†^				*P* value^‡^
	< 7.5	7.5 to < 22.5	22.5 to < 37.5	≥ 37.5	
Participants, n	4832	8600	4329	8823	
Male, n (%)	1904 (39.4)	3578 (41.6)	1855 (42.9)	4143 (47.0)	< 0.001
Age, years	62.9 (9.2)	63.7 (8.4)	63.2 (8.2)	63.3 (7.9)	< 0.001
**Education, n (%)**					< 0.001
Primary school or below	1072 (22.2)	1703 (19.8)	882 (20.4)	1729 (19.6)	
Middle school	1773 (36.7)	3105 (36.1)	1561 (36.1)	3398 (38.5)	
High school or beyond	1954 (40.4)	3745 (43.6)	1863 (43.0)	3650 (41.4)	
**Smoking status, n (%)**					< 0.001
Never	3491 (72.3)	6303 (73.3)	3153 (72.8)	6338 (71.8)	
Former	363 (7.5)	840 (9.8)	463 (10.7)	1001 (11.4)	
Current	969 (20.1)	1437 (16.7)	703 (16.2)	1465 (16.6)	
**Drinking status, n (%)**					
Never	3535 (73.2)	6104 (71.0)	3022 (69.8)	5886 (66.7)	< 0.001
Former	181 (3.8)	372 (4.3)	211 (4.9)	458 (5.2)	
Current	1102 (22.8)	2106 (24.5)	1082 (25.0)	2464 (27.9)	
**Consumption of foods (≥ 5 times/week, %)**					
Meat	1941 (40.2)	3432 (39.9)	1690 (39.0)	3549 (40.2)	0.622
Fruits	2456 (50.8)	4926 (57.3)	2506 (57.9)	5465 (61.9)	< 0.001
Vegetables	4568 (94.5)	8269 (96.2)	4160 (96.1)	8487 (96.2)	< 0.001
BMI, kg/m^2^	24.1 (3.3)	24.1 (3.2)	23.9 (3.1)	24.0 (3.0)	0.002
Hypertension, yes (%)	2538 (52.5)	4920 (57.2)	2388 (55.2)	5057 (57.3)	< 0.001
Hyperlipidemia, yes (%)	1782 (36.9)	3405 (39.6)	1674 (38.7)	3328 (37.7)	0.008
Diabetes, yes (%)	731 (15.1)	1534 (17.8)	683 (15.8)	1468 (16.6)	< 0.001
Family history of CVD, n (%)	607 (12.6)	1128 (13.1)	571 (13.2)	1168 (13.2)	0.708
LTPA (MET-hours/week)	1.4 (2.3)	16.6 (4.7)	30.0 (3.9)	67.3 (37.0)	< 0.001
Total sedentary time (hours/week)	23.7 (15.1)	22.3 (12.2)	22.5 (12.2)	22.3 (12.0)	< 0.001
Screen activities, (hours/week)	20.6 (12.9)	19.6 (10.3)	20.0 (10.6)	19.6 (10.1)	< 0.001
Playing Mahjong, (hours/week)	3.1 (7.4)	2.8 (6.5)	2.6 (6.1)	2.7 (6.4)	< 0.001

BMI, body mass index; MET, metabolic equivalent of task.

Data are means  (SD) for continuous variables or number (percentages) for categorical variables. Missing data were 149, 58, 61, 2814, 264, 301, 224, 230, 26 and 241 for education, smoking status, drinking status, BMI, meat, fruits, vegetables, Mahjong, TV and total sedentary time.

^†^< 7.5, 7.5–22.5, 22.5–37.5, and ≥ 37.5 MET-hours/week represented < 1, 1–3, 3–5, and ≥ 5 times of the minimum recommended LTPA level, respectively.

^‡^*P* values were derived from one-way analysis of variance (ANOVA) for continuous variables, and Chi-square test for the categorical variables.

### Association of LTPA with incident CVD

During a mean 5.0 (1.5) years of follow-up, we documented 5704 incident CVD cases, including 4659 incident CHD cases and 1045 incident stroke cases. Table 2 presented the association between LTPA and incident CVD. Compared with participants reporting LTPA less than the recommended minimum of 7.5 MET-hours/week, an 18% lower risk (HR, 0.82 [95% CI 0.75 to 0.91]) was seen for those with 3 to 5 times the recommended minimum (22.5–37.5 MET-hours/week); however, no significant additional benefit was gained when exceeding 5 times the recommended minimum (> 37.5 MET-hours/week; HR, 0.81 [95% CI 0.73 to 0.90]). The adjusted HRs (95% CIs) for CVD across categories of LTPA (7.5 to ≤ 22.5, 22.5 to ≤ 37.5, > 37.5 MET-hours/week) were 0.93 (0.86 to 1.01), 0.82 (0.75 to 0.91) and 0.81 (0.73 to 0.90), respectively (*P*_trend_ < 0.001). Similar associations were observed for both CHD (*P*_trend_ = 0.006) and stroke (*P*_trend_ = 0.001). Sensitivity analysis by extended adjustment or further exclusions did not appreciably alter the association (Supplementary Table S1 online). In stratified analysis, we found that the result was more obvious in male (HR 0.80 [95%CI 0.73 to 0.87]; *P*_interaction_ = 0.02); and the cardiovascular benefits were consistent across age, education, smoking status, drinking status, BMI, hypertension, hyperlipidemia and diabetes. (*P*_interaction_ > 0.05; Supplementary Fig. S1 online).

**Table 2:** The association of leisure-time physical activity with cardiovascular disease.

	Leisure-time physical activity, MET-hours/week^∗^				*P* trend
	< 7.5	7.5 to < 22.5	22.5 to < 37.5	≥ 37.5	
**Incident CVD**					
Cases/person years	1112/23,581	1939/42,539	857/21,780	1796/44,495	
Model 1^†^	Ref.	0.93 (0.87 to 1.00)	0.82 (0.75 to 0.90)	0.84 (0.78 to 0.91)	< 0.001
Model 2^‡^	Ref.	0.93 (0.86 to 1.01)	0.82 (0.75 to 0.91)	0.81 (0.73 to 0.90)	< 0.001
**Incident CHD**					
Cases/person years	876/24,079	1585/43,352	702/22,132	1496/45,211	
Model 1	Ref.	0.98 (0.90 to 1.06)	0.87 (0.79 to 0.96)	0.91 (0.84 to 0.99)	0.010
Model 2	Ref.	0.97 (0.89 to 1.05)	0.86 (0.77 to 0.96)	0.86 (0.77 to 0.97)	0.006
**Incident stroke**					
Cases/person years	236/25,744	354/46,648	155/23,653	300/48,344	
Model 1	Ref.	0.78 (0.67 to 0.92)	0.69 (0.56 to 0.85)	0.64 (0.54 to 0.76)	< 0.001
Model 2	Ref.	0.81 (0.68 to 0.97)	0.72 (0.58 to 0.90)	0.65 (0.50 to 0.83)	0.001

∗< 7.5, 7.5–22.5, 22.5–37.5, and ≥ 37.5 MET-hours/week represented < 1, 1–3, 3–5, and ≥ 5 times of the minimum recommended LTPA level, respectively.

^†^Adjusted for age and sex.

^‡^Additionally adjusted for education, smoking status, alcohol intake, consumption of food (meat, vegetables, and fruit), hypertension, hyperlipidemia, diabetes, BMI, MET-hours/week, total sedentary time, and family history of CVD.

**Figure 1:**
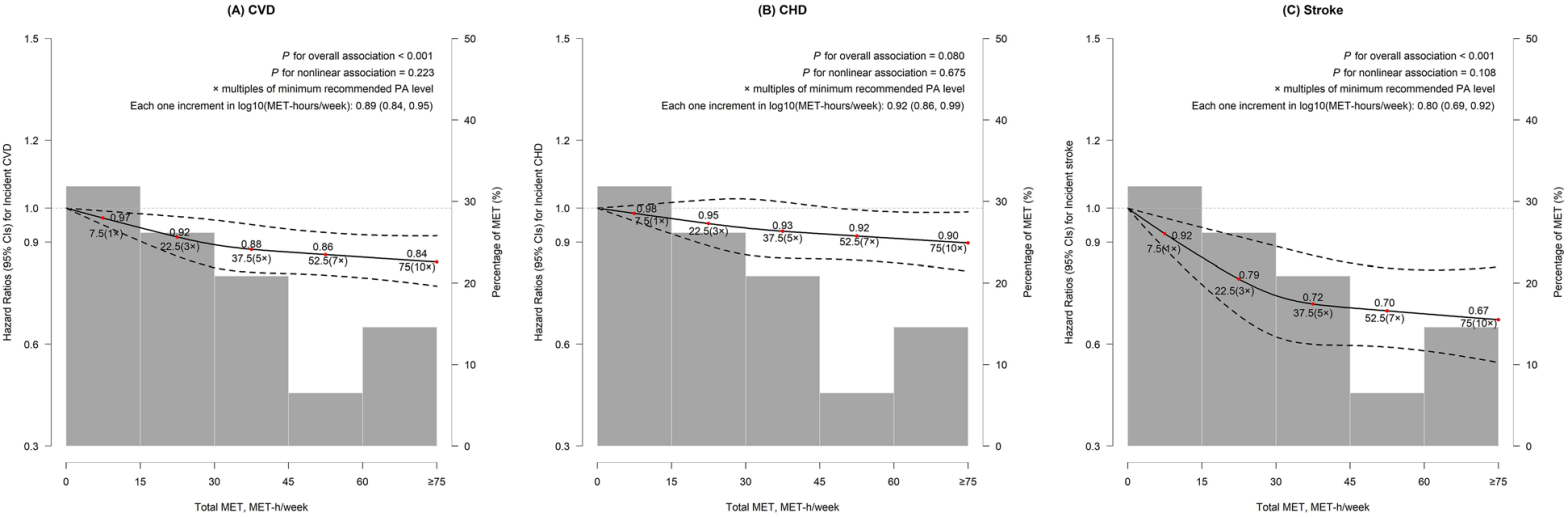
Adjusted hazard ratios for CVD, stroke and CHD according to levels of leisure-time physical activity. (**A**) Association between leisure-time physical activity and incident CVD. (**B**) Association between leisure-time physical activity and incident CHD. (**C**) Association between leisure-time physical activity and incident stroke. The multivariable models were adjusted for age, sex, education, smoking status, alcohol intake, consumption of food (meat, vegetables, and fruit), hypertension, hyperlipidemia, diabetes, BMI, total sedentary time, and family history of CVD.

Cubic spline analysis showed that any level of LTPA was associated with significantly lower risks of CVD and stroke (*P*_CVD_ < 0.001, Fig. 1A; *P*_stroke_ < 0.001, Fig. 1C). Each one increment in log_10_ (MET-hours/week) was associated with 11%, 8%, and 20% lower risks of CVD (HR 0.89 [95% CI 0.84 to 0.95]), CHD (HR, 0.92 [95% CI 0.86 to 0.99]), and stroke (HR 0.80 [95% CI 0.69 to 0.92]), respectively (Fig. 1). Compared with no baseline LTPA, the most rapid decrease in CVD risk was seen before 3 times the WHO recommended minimum of PA. For total LTPA, the risks decreased by 8% for CVD (95% CI 2 to 14%) and 21% for stroke (95% CI 8 to 32%), respectively at 3 times the recommended minimum.

### Association between different types of LTPA and incident CVD

**Figure 2:**
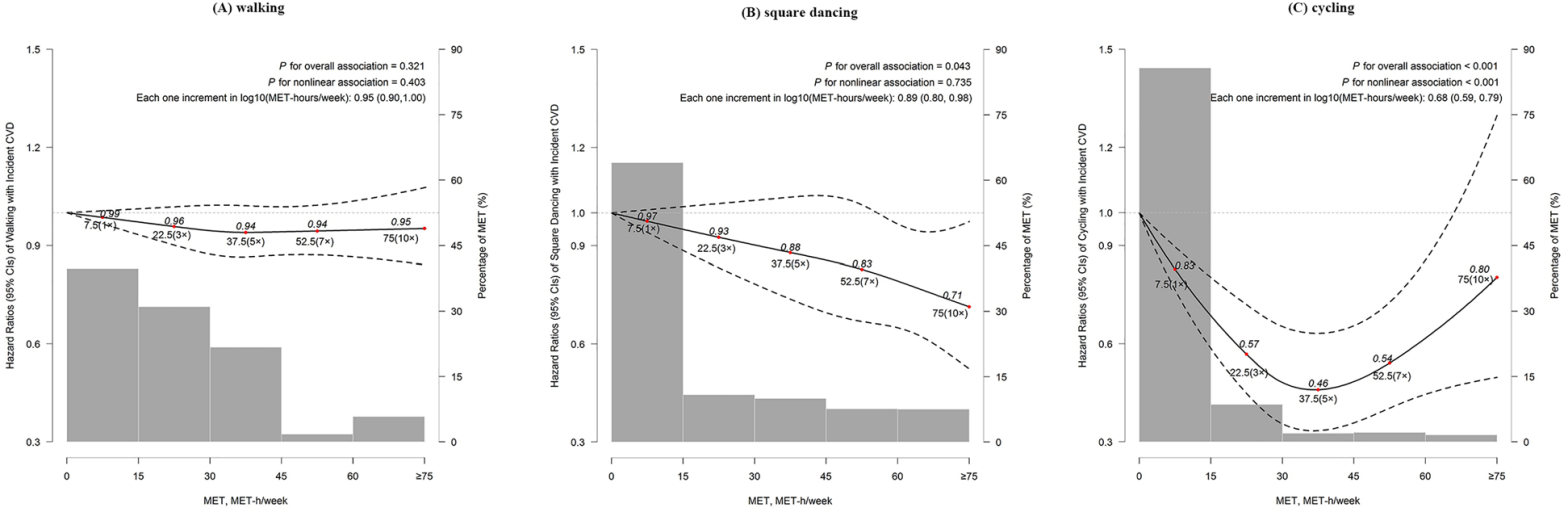
Adjusted hazard ratios for CVD according to levels of different types of leisure-time physical activity. (**A**) Association between walking and incident CVD. (**B**) Association between square dancing and incident CVD. (**C**) Association between cycling and incident CVD. The multivariable models were adjusted for age, sex, education, smoking status, alcohol intake, consumption of food (meat, vegetables, and fruit), hypertension, hyperlipidemia, diabetes, BMI, total sedentary time, other LTPA, and family history of CVD.

When examining specific types of LTPA, a monotonic decrease in CVD risk was seen with increasing levels of square dancing (Each one increment in log_10_ [MET-hours/week]: HR, 0.89 [95% CI 0.80 to 0.98]; Fig. 2B), and such association was mainly restricted to female (*P* = 0.014; Supplementary Fig. S2 online). However, For cycling, we identified a U-shaped curve for the association, with the maximum observed cardiovascular benefit accrued with 37.5 MET-hours/week, equivalent to about 9 h cycling per week (HR, 0.46 [95% CI 0.33 to 0.63]); Fig. 2C). No significant association was seen for other types of LTPA (Fig. 2 and Supplementary Fig. S3 online).

### Interaction between LTPA and sedentary

**Figure 3:**
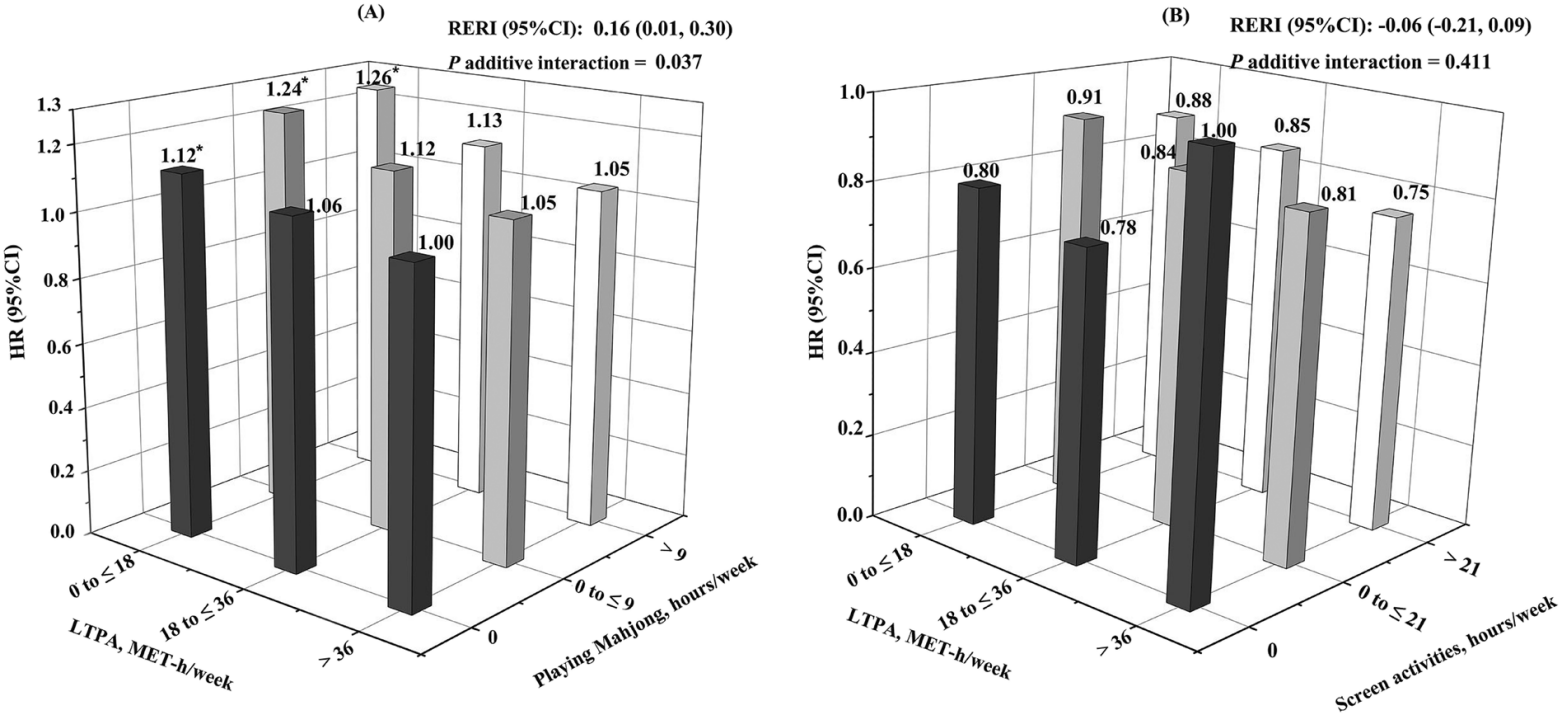
The joint effect of LTPA and sedentary behavior on incident CVD. (**A**) The joint effect of LTPA and Mahjong playing on incident CVD. (**B**) The joint effect of LTPA and screen activities on incident CVD. The multivariable models were adjusted for age, sex, education, smoking status, alcohol intake, consumption of food (meat, vegetables, and fruit), hypertension, hyperlipidemia, diabetes, BMI, MET-hours/week, and family history of CVD. *P < 0.05. ^†^The number was 26,354 for Mahjong and incident CVD analysis; the number was 26,558 for screen activities and incident CVD analysis. ^‡^The hazard ratios (95% CI) from the most active to the least active group were: 1 (reference), 1.06 (1.00 to 1.16), 1.12 (1.01 to 1.24), 1.05 (0.90 to 1.21), 1.12 (0.96 to 1.30), 1.24 (1.07 to 1.44), 1.05 (0.90 to 1.21), 1.13 (0.97 to 1.32), 1.26 (1.08 to 1.47), respectively, in (**A**). ^¶^The hazard ratios (95% CI) from the most active to the least active group were: 1 (reference), 0.78 (0.45 to 1.35), 0.80 (0.51 to 1.25), 0.81 (0.57 to 1.14), 0.84 (0.59 to 1.19), 0.91 (0.64 to 1.30), 0.75 (0.53 to 1.07), 0.85 (0.60 to 1.22), 0.88 (0.61 to 1.25), respectively, in (**B**).

Participants reported prolonged time spent playing Mahjong have a significantly higher risk of incident CVD in comparison with those not playing (HR comparing extreme categories, 1.09 [95% CI 1.00 to1.18]; *P*_trend_ = 0.022), whereas no significant association was observed for screen activities (HR comparing extreme categories, 0.98 [95% CI 0.81 to1.20]; Supplementary Table S2 online). In addition, our findings showed significant additive interactions of LTPA and playing Mahjong for incident CVD (*P*_additive interaction_ = 0.037; Fig. 3A), no significance was found for screen activities (*P*_additive interaction_ = 0.411; Fig. 3B). Compared with the most active participants (i.e., those with more than 36 MET-hours/week), those in the bottom LTPA tertile had a significantly higher risk of CVD, regardless of time spent playing Mahjong. No such association was seen for screen activities.

## Discussion

In this prospective study of Chinese retired adults, our results supported that meeting the WHO recommended minimum was associated with significantly lower CVD risk and further suggested the optimal benefit by reaching 3 to 5 times the recommended minimum. Encouraging retired adults to participate in square dancing and cycling might gain favourable cardiovascular benefits. Interestingly, we found that those with high LTPA level might mitigate the excess risk of CVD associated with sedentary behaviour, particularly playing Mahjong.

Our finding of reduced CVD risk associated with higher LTPA level was consistent with previous studies^24–28^. A historically prospective cohort study of 416,175 healthy individuals from Taiwan found that, compared with inactive individuals, those who were meeting PA recommendations had a 32% lower risk of CVD mortality (HR, 0.68 [95% CI 0.61 to 0.76])^29^. A pooled analysis (661,137 participants, about 14.2 years follow-up) found the benefit threshold of a 42% lower risk of CVD mortality (HR, 0.58 [95% CI 0.56 to 0.61]) with LTPA of 3 to 5 times the recommended minimum (22.5–40.0 MET-hours/week) compared with no LTPA^30^. Furthermore, another study of 88,140 US adults indicated that no additional cardiovascular health benefit was found when doing more than 10 times the recommended LTPA^31^. Despite focusing on working adults, these studies investigated only fatal CVD, and the present study of retired adults further extended previous findings by showing appreciable benefits related to incident CVD when exceeding the WHO recommendations. The beneficial effect in male might because that the LTPA level was higher than that in female (mean [SD]: 35 [36] in male and 31 [32] in female; *P* < 0.001). The relatively lower risk of stroke associated with LTPA observed in our study was in line with the effect of walking in the cohort study with 73,265 participants^32^ and another Japanese study with 74,913 participants^25^, which might be due to the better improvement of cerebral blood flow and perfusion through LTPA^33^.

The interesting finding was that square dancing was associated with a lower CVD risk. A cross-sectional study of 1944 adults in China found a 30% lower prevalence of CHD among adults with moderate or high frequency in walking or square dancing^34^. To date, only one prospective cohort study of 48,390 British adults, reported that dancing was associated with a 46% lower risk of cardiovascular mortality during a mean of 9.7 years of follow-up^35^. The present study found that a monotonic decrease in CVD risk with increasing levels of square dancing. Despite the different outcomes (CVD mortality versus incidence) investigated, the lacked consistency in the magnitude of CVD benefits could be interpreted by the between-racial differences in intensity of dance performed in China and western countries. Square dancing, a popular LTPA type in China, can be easily accepted by low-income people^36^. The superior benefits of square dancing over other types of leisure activity may be due to the lessened daily mental stress through its direct social and entertaining components^37^. To our knowledge, the present study was the first prospective study to explore the association between square dancing and incident CVD among retired adults, suggesting that promoting participation in this new kind of leisure activity might be a prioritized strategy for CVD prevention. In addition, the benefit of cycling was similar with the results in UK biobank, which might derive from the relatively higher intensity of cycling^38^.

Notably, this study firstly identified that a higher risk of CVD among retired adults who played Mahjong during leisure times, and found that exceeding 36 MET-hours/week of LTPA, which was roughly equivalent to 8 h/week of moderate-intensity PA, could eliminate the adverse effect of playing Mahjong. In line with our finding, a recent prospective study of 149,077 participants identified elimination of the adverse sitting effects in the group with over 35.5 MET-hours/week of PA^39^. Similarly, a harmonized meta-analysis of over 1 million individuals also found that 7–9 h of moderate-intensity PA per week might eliminate the elevated mortality risks associated with prolonged sitting time^40^. Moreover, we observed that the effect of playing mahjong on CVD incidence seemed to be stronger in magnitude than that in screen activities. It is plausible that playing mahjong typically occurs in the card room, which might be accompanied by high exposure of second-hand smoke, thus increasing CVD risk^41^. These findings together highlight the importance of active participation in LTPA in CVD prevention, especially among those with prolonged sedentary time.

The major strengths of the present study are the prospective design, high response rates for baseline and follow-up surveys, and inclusion of participants after retirement, which minimizes confounding from other domains of PA including occupation and transportation. To our best knowledge, this is the first prospective study reported the inverse association of square dancing with incident CVD among Chinese retired adults. The present study had certain limitations. First, as with most large population studies, information of LTPA in the present study was self-reported at baseline, and the LTPA change during the follow-up period was not accounted. However, misclassification might be random and thus would bias the association towards the null^42^. Second, the possibility of reverse causality cannot be completely ruled out in the present study^43^; however, we excluded CVD and cancer at baseline, and CVD cases occurring during the first year of follow-up to address the issue of reverse causation. Finally, despite carefully controlling for a variety of covariates, residual confounding remains possible.

## Conclusions

Higher LTPA levels were associated with a lower risk of CVD, a benefit threshold for CVD prevention was achieved at 3 to 5 times the recommended PA minimum. Encouraging retired adults to participate in square dancing and cycling might gain favourable cardiovascular benefits. These data will be informative for future updates for guidelines with respect to the appropriate amount and type of leisure activity for CVD prevention.

## Methods

### Study population

The present study was based on the Dongfeng-Tongji (DFTJ) cohort, which has been reported previously^44^. Briefly, the DFTJ cohort is an ongoing prospective cohort in Shiyan, Hubei, China, which was established in 2008 and recruited retired employees of the Dongfeng Motor Corporation (DMC) quinquennially. The present study enrolled 38,295 participants from the survey from April to October 2013, and baseline information of whom was collected by questionnaire and physical examination. After excluding 10,254 participants with coronary heart disease (CHD, n = 6457), stroke (n = 2406), severely abnormal electrocardiogram (n = 838) and cancer (n = 2686) in 2013, 1402 participants with missing data for LTPA and 55 participants reported implausible information on LTPA, and leaved 26,584 participants in the final analysis. The flowchart of study population was presented in Supplementary Fig. S4 online.

### Assessment of leisure-time physical activity and sedentary behaviour

LTPA and sedentary behaviour were assessed through the questionnaire in 2013. For those reporting participation in LTPA at least twice per month, we multiplied the MET intensity^45,46^ by frequency and time spent on this activity and summed across activities to estimate overall LTPA energy expenditure in MET-hours/week. Detailed information was listed in Supplementary Table S3 and eMethods online.

### Assessment of covariates

Covariates comprised age, sex (male/female), education (primary or below, middle, high school or beyond), body mass index (BMI), smoking status (current, former, never), alcohol intake status (current, former, never), hypertension (yes/no), hyperlipidemia (yes/no), diabetes (yes/no), consumption frequency of meat, vegetables, fruit (≥ 5 times/week), LTPA (MET-hours/week), total sedentary time (hours/week) and family history of CVD (yes/no), which were measured at baseline. Further details on the assessment of covariates are provided in eMethods.

### Ascertainment of outcome

The diagnosis of CVD was based on International Classification of Disease (ICD) codes of the World Health Organization (10th revision) by clinicians of Sinopharm Dongfeng General Hospital through medical insurance system and the medical record reviews^47^. The outcome of interest in the present study was incident CVD, including CHD and stroke^48^ that occurred from baseline survey until December 31, 2018. Incident CHD was defined as the first hospital admission with an occurrence of angina pectoris (ICD-10 code I20), acute myocardial infarction (AMI, I21), subsequent myocardial infarction (I22), other forms of acute (I24) or chronic (I25) heart disease, percutaneous transluminal coronary angioplasty or coronary artery bypass graft, and cardiac arrest (I46) or death with CHD (I20-I25) as the underlying cause during follow-up^49^. Incident stroke (I60-I61, I63-I64, I69.0-I69.1, and I69.3-I69.4) was defined as the first sudden or rapid onset of a typical neurological deficit caused by vascular origin over 24 h or until death^47^.

### Statistical analysis

Because of the skewed distribution of calculated MET-hours/week, data of LTPA was logarithmically transformed before statistical analysis (log_10_ [MET-hours/week]). We also created four categories for calculated MET-hours/week: < 7.5, 7.5 to < 22.5, 22.5 to < 37.5, ≥ 37.5 MET-hours/week, to reflect multiples of the 2010 PA recommendations from WHO, equivalent to insufficient LTPA, 1–3 times, 3–5 times, and 5 or more times the minimum recommended level^5,30^. Cox-proportional hazard models were used to estimate hazard ratios (HRs) and 95% confidence intervals (CIs) for the association of LTPA with the risk of incident CVD with adjustments for age and sex in model 1, and additionally adjusted for education, BMI, smoking status, alcohol intake status, hypertension, hyperlipidemia, diabetes, consumption of food (meat, vegetables, fruit), MET-hours/week, total sedentary time and family history of CVD in model 2. Test for linear trends across categories of LTPA was derived by assigning the median value for each category.

The restricted cubic spline was used to delineate the dose–response relationship of LTPA levels with incident CVD, CHD and stroke. Those reporting more than the 90th percentile of total LTPA were assigned a value of 75 MET-hours/week (n = 2316)^50^. In addition, we further examined whether the dose–response association between LTPA and CVD risk differed across participants with different types of LTPA.

We also applied stratification analysis for association between LTPA and incident CVD according to several potential confounders at baseline. To evaluate the potential modification by subgroups, we used the Wald test for dichotomous variables and the likelihood ratio test for multi-level variables for interaction between LTPA and subgroups variable^51^. We also assessed the joint association of LTPA with sedentary behaviour by creating combined exposures, using those who were the most physically active (> 36 MET-hours/week of LTPA plus 0 h/week for Mahjong or 0 h/week for screen activities, respectively) as the reference group. The significance of additive interaction was indicated by a value of the relative excess risk due to interaction greater than zero^52^.

To test the robustness of results, we performed sensitivity analysis by extended adjustment for household activity, excluding participants reporting over 6 h of LTPA per day (n = 189), excluding participants retired 5 years before the mandatory age for retirement (n = 5882; 55 years for male and 50 years for female), or excluding CVD cases occurred during the first year of follow-up (n = 665).

All statistical analyses were performed using the SAS 9.4 software package (SAS Institute, Cary, North Carolina, USA).

### Ethics approval and consent to participate

The study was approved by the Ethics Committee of School of Public Health, Tongji Medical College, Huazhong University of Science and Technology (2012-10). All participants provided written informed consent to partake in the study.

### Consent for publication

All participants were consent for publication.

## Supplementary Information

Supplementary Information.

## Data Availability

The data of this study are available from the corresponding author upon reasonable request.
