# Supplementary material for: Leisure-time physical activity and risk of incident cardiovascular disease in Chinese retired adults
Source: Sci Rep. 2021 Dec 17;11:24202. doi: 10.1038/s41598-021-03475-6 (PMC8683485; doi:10.1038/s41598-021-03475-6)
Supplement: Supplementary file 1 — Supplementary Information. [file 41598_2021_3475_MOESM1_ESM.pdf]

Leisure-time physical activity and risk of incident cardiovascular disease in Chinese retired adults

Xuanwen Mu[1†]; Kuai Yu[1†]; Pinpin Long[1]; Rundong Niu[1]; Wending Li[1]; Huiting Chen[1]; Hui Gao[1]; Xingxing Li[1]; Yu Yuan[1]; Handong Yang[2]; Xiaomin Zhang[1]; Mei-an He[1]; Gang Liu[3]; Huan Guo[1]; Tangchun Wu[1*].

[1]Department of Occupational and Environmental Health, Key Laboratory of Environment and Health, Ministry of Education and State Key Laboratory of Environmental Health (Incubating) School of Public Health, Tongji Medical College, Huazhong University of Science and Technology, 430030 Wuhan, China.

[2]Department of Cardiovascular Diseases, Sinopharm Dongfeng General Hospital, Hubei University of Medicine, Shiyan 442000, China.

[3]Department of Nutrition and Food Hygiene, School of Public Health, Tongji Medical College, Huazhong University of Science and Technology, Wuhan 430030, China.

[†] These authors contributed equally to the study.

[*] **Corresponding author:**

Tangchun Wu, MD, PhD

Professor, Department of Occupational and Environmental Health

Director, State Key Laboratory of Environmental Health for Incubation

Dean, School of Public Health

Vice Dean, Tongji Medical College

Huazhong University of Science and Technology

13 Hangkong Rd, Wuhan 430030, Hubei, China.

Phone: 86-27-83692347; Fax: 86-27-83692560

wut@mails.tjmu.edu.cn

eMethods 1

Supplementary Table S1. Adjusted hazard ratios for CVD associated with leisure-time physical activity after extended adjustments or further exclusions.. 6

Supplementary Table S2. Association between sedentary behaviour and incident CVD. 8

Supplementary Table S3. Leisure-time physical activity types, MET values, codes and specific activities. 9

Supplementary Figure S1. Association of LTPA with incident CVD stratified by potential modifiers. 10

Supplementary Figure S2. The restricted cubic spline for the association of square dancing with incident CVD stratified by gender. 11

Supplementary Figure S3. Adjusted hazard ratios for CVD according to levels of different types of leisure-time physical activity. 12

Supplementary Figure S4. The flowchart of study participants. 13

eMethods

Assessment of leisure-time physical activity and sedentary behaviour

LTPA and sedentary behaviours were assessed through the following questions from the baseline questionnaire. For assessment of LTPA, we asked participants: “In the past year, how often did you engage in the following activities and what was the duration?” (Multiple options including no LTPA, walking, square dancing, cycling, jogging, ball, Tai chi, public facility, mountain, conditioning exercise and swimming). Participants who reported to participate at least one type of LTPA were further asked the corresponding frequency and average time per session for each LTPA. For those reporting participation in LTPA for at least twice per month, we calculated the weekly metabolic equivalent of task-hours ([MET]-hours/week) by multiplying the MET intensity (3 for walking, 3.8 for conditioning exercise, 4 for cycling, 4.5 for Tai chi, 5 for square dancing, 6 for ball or public facility, 6.3 for mountain and 7.5 for jogging or swimming; Supplementary Table S3) [1, 2] by frequency and time spent on this activity and summed across activities to estimate overall LTPA energy expenditure in MET-hours/week. In order to get an accurate MET intensity, we looked up the specific MET intensity in the 2011 Compendium of Physical Activities and calculated relevant weights (Supplementary Table S3). For example, ball games were assigned 1/3 weights by calculating the mean MET intensity of the specific LTPA types (basketball, tennis, soccer). “missing data” for LTPA was defined as missing one of the follow questions: 1 Type of LTPA; 2 Frequency of LTPA; 3 Duration of LTPA. Implausible information for LTPA was those participating LTPA for over 12 hours per

day or reporting no LTPA, but values>0 in the frequency or duration variables of at least one type of LTPA.

For assessment of sedentary behaviour, each participant was asked to provide their daily average time spent sitting at home on screen activities, including watching TV, VCR, and computer. We also asked participants: “In the past year, how many times per week did you play Mahjong?” Participants who reported at least once per week were further asked: “On average, about how many hours each time did you play Mahjong?”. The weekly average time spent on playing Mahjong was calculated by multiplying frequency and time spent on playing Mahjong.

Assessment of covariates

Weight-related indicators were measured in light clothing without shoes. Body mass index (BMI) was calculated as weight (kilogram) divided by height (meter) squared. Current smokers were defined as smoking at least one cigarette per day for more than half a year. Current drinkers were defined as drinking alcohol at least one time per week for more than half a year. Family history of CVD including CHD and stroke was limited to first-degree family members. Hypertension was defined as being on anti-hypertensive medication, a systolic blood pressure >= 140 mmHg, a diastolic blood pressure >= 90 mmHg or a self-reported physician diagnosis. Hyperlipidemia was defined as total cholesterol >= 6.22 mmol /L, triglycerides >= 2.26 mmol/L, High-density lipoprotein cholesterol (HDL-C) < 1.04 mmol/L, low-density lipoprotein cholesterol (LDL-C) >= 4.14 mmol/L, medication use or a previous physician diagnosis. Diabetes was defined by fasting glucose >= 7.0 mmol/L or

haemoglobin A1c (HbA1c) >= 6.5% or self-reported use of anti-diabetic medications (oral hypoglycemic medication or insulin). An overnight fast blood sample was collected by Sinopharm Dongfeng hospital. All biochemical blood indicators were measured using ARCHITECT ci8200, Abbott, USA. Missing values were replaced by mean or median, furtherly adjusted for dichotomous variables.

Detailed statistical analysis

The present study excluded 10254 participants with CHD (n=6457), stroke (n=2406), and severely abnormal electrocardiogram (n=838; severe abnormal electrocardiogram including atrial fibrillation, atrial flutter, pre-excitation syndrome, pacemaker rhythm, frequent premature ventricular contractions)[3] and cancer (n=2686) in 2013, we excluded 1402 participants with missing data for physical activity and 55 participants reported implausible information on physical activity, and leaved 26584 participants in the final analyses (Supplementary Fig. S4). Basic characteristics were presented as mean (standard deviation) if under normal distribution and median (interquartile) if under skew distribution for continuous variables, and categories variables according to multiples of the minimum recommended LTPA level (Table 1). Cox proportional hazards regression models were used to calculate HRs and 95% confidence intervals (CIs) for cardiovascular diseases associated with baseline LTPA (Table 2). Analyses were adjusted for age and sex (model 1) and additionally adjusted for education, BMI, smoking status, alcohol intake status, hypertension, hyperlipidemia, diabetes, diet frequency (meat, fruit, vegetables), MET-hours/week, total sedentary time and family history of CVD (model

2). The reference group came from those below the minimum WHO recommended physical activity level (7.5 MET-hours/week).

The restricted cubic spline with 3 knots (25th, 50th and 75th) was used to explore the dose-response relationship between LTPA amount and incident CVD (Fig. 1, Fig2, and Supplementary Fig. S2, S3). The cutoff values were 75 MET-hours/week (about 90% total LTPA amount winsorized).

Stratification analyses for associations between LTPA and incident CVD according to several potential confounders were presented in the Supplementary Fig. S1. In addition, the dose-response analyses of square dancing stratified by gender were presented in Supplementary Fig. S2.

We also explored the additive interaction of LTPA and sedentary behaviour (including screen activities and playing Mahjong), using those who were the most physically active (> 36 MET-hours/week of LTPA plus 0 hours/week for Mahjong or 0 hours/week for screen activities) as the reference group (Fig. 3). The risk of sedentary behaviour with CVD was presented in Supplementary Table S2 categorized by none and median of sedentary behaviour time.

Sensitivity analyses were conducted by extended adjustment for household activity; excluding participants reporting over 6 hours of LTPA per day (n=189); excluding CVD cases (n=665) occurred during the first year of follow-up; excluding participants retired 5 years before the mandatory age for retirement (n=5882; 55 years for male and 50 years for female; Supplementary Table S1).

All statistical analyses were performed using the SAS 9.4 software package

(SAS Institute, Cary, North Carolina, USA). A two-sided test and $P < 0.05$ was considered statistically significant.

Supplementary Table S1. Adjusted hazard ratios for CVD associated with leisure-time physical activity after extended adjustments or further exclusions.

Events	No. of total events	Main model *	With extended adjustment †	Excluding participants reporting over 6 hours per day of LTPA (n = 189)	Excluding CVD cases occurred during the first year of follow-up (n = 665)	Excluding participants retired 5 years before statutory retirement age (male < 55 years and female < 50 years; n = 5882)
CVD						
< 7.5	1112 (19%)	Ref.	Ref.	Ref.	Ref.	Ref.
7.5 to < 22.5	1939 (34%)	0.93 (0.86 to 1.01)	0.94 (0.87 to 1.01)	0.95 (0.88 to 1.02)	0.95 (0.88 to 1.03)	0.97 (0.89 to 1.06)
22.5 to < 37.5	857 (15%)	0.82 (0.75 to 0.91)	0.83 (0.76 to 0.92)	0.85 (0.77 to 0.94)	0.84 (0.76 to 0.93)	0.88 (0.79 to 0.98)
≥ 37.5	1796 (31%)	0.81 (0.73 to 0.90)	0.83 (0.74 to 0.92)	0.86 (0.76 to 0.97)	0.84 (0.75 to 0.95)	0.88 (0.77 to 1.00)
CHD						
< 7.5	876 (19%)	Ref.	Ref.	Ref.	Ref.	Ref.
7.5 to < 22.5	1585 (34%)	0.97 (0.89 to 1.05)	0.97 (0.89 to 1.06)	0.99 (0.91 to 1.08)	1.01 (0.92 to 1.10)	0.99 (0.90 to 1.09)
22.5 to < 37.5	702 (15%)	0.86 (0.77 to 0.96)	0.87 (0.78 to 0.97)	0.90 (0.80 to 1.00)	0.88 (0.79 to 0.99)	0.90 (0.80 to 1.01)
≥ 37.5	1496 (32%)	0.86 (0.77 to 0.97)	0.89 (0.79 to 1.01)	0.95 (0.83 to 1.08)	0.91 (0.80 to 1.04)	0.92 (0.80 to 1.05)
Stroke						
< 7.5	236 (23%)	Ref.	Ref.	Ref.	Ref.	Ref.
7.5 to < 22.5	354 (34%)	0.81 (0.68 to 0.97)	0.81 (0.68 to 0.96)	0.80 (0.67 to 0.96)	0.77 (0.64 to 0.92)	0.90 (0.73 to 1.10)
22.5 to < 37.5	155 (15%)	0.72 (0.58 to 0.90)	0.71 (0.57 to 0.89)	0.70 (0.56 to 0.88)	0.71 (0.56 to 0.90)	0.84 (0.65 to 1.08)
≥ 37.5	300 (29%)	0.65 (0.50 to 0.83)	0.63 (0.49 to 0.81)	0.61 (0.46 to 0.81)	0.61 (0.46 to 0.80)	0.76 (0.56 to 1.04)

* Hazard ratios were adjusted for age, sex, education, smoking status, alcohol intake, consumption of food (meat, vegetables, and fruit), hypertension,

hyperlipidemia, diabetes, BMI, MET-hours/week, total sedentary time, family history of CVD.

† Additionally adjusted for housework, MET-hours/day.

Supplementary Table S2. Association between sedentary behaviour and incident CVD.

	Cases/person years	HR (95% CI)	*P* trend
Playing Mahjong [*] [†] [‡]			
None	4308/99978	Ref.	0.022
≤ 9 hours/week	686/16468	1.08 (1.00 to 1.17)	
> 9 hours/week	667/14798	1.09 (1.00 to 1.18)	
Screen activities [*] [†] [‡]			
None	105/2176	Ref.	0.350
≤ 21 hours/week	4096/94966	1.01 (0.83 to 1.23)	
> 21 hours/week	1498/35122	0.98 (0.81 to 1.20)	

[*] Categorized according to the median of each sedentary behaviour.

[†] The multivariable models were adjusted for age, sex, education, smoking status, alcohol intake, consumption of food (meat, vegetables, and fruit), hypertension, hyperlipidemia, diabetes, BMI, MET-hours/week, and family history of CVD.

[‡] The number was 26354 for Mahjong and incident CVD analysis; 26558 for screen activities and incident CVD analysis.

Supplementary Table S3. Leisure-time physical activity types, MET values, codes and specific activities.

**Activity types**	**MET**	**Codes**	**Specific Activities**
Ball games	6	Mean of 15050 15695 15610	Basketball, tennis, soccer
Cycling	4	1010	Bicycling, leisurely, for pleasure
Conditioning exercise	3.8	Mean of 15300 02064 02022	Home exercise, general, calisthenics
Jogging	7.5	Mean of 12020 12025	General jogging, jogging in place
Mountain	6.3	17033	Climbing hills, no load
Public facility	6	Mean of 2090 2050	Slinmastics, jazzercise, resistance training, power lifting of body building
Square dancing	5	Mean of 3030 3025	Cultural dancing, ballroom
Swimming	7.5	Mean of 18280 18300 18310 18330	Fast, swim in the lake, leisurely, synchronized
Tai chi	4.5	Mean of 15670 17080	Tai chi, qi gong, general, hiking
Walking	3	17170	Walking, level, firm surface

MET: Metabolic equivalent of task.

Based on the 2011Compendium of Physical Activities: a second update of codes and MET values. Ainsworth BE, et al. Medicine and Science in Sports and Exercise, 2011; 43(8):1575-1581.

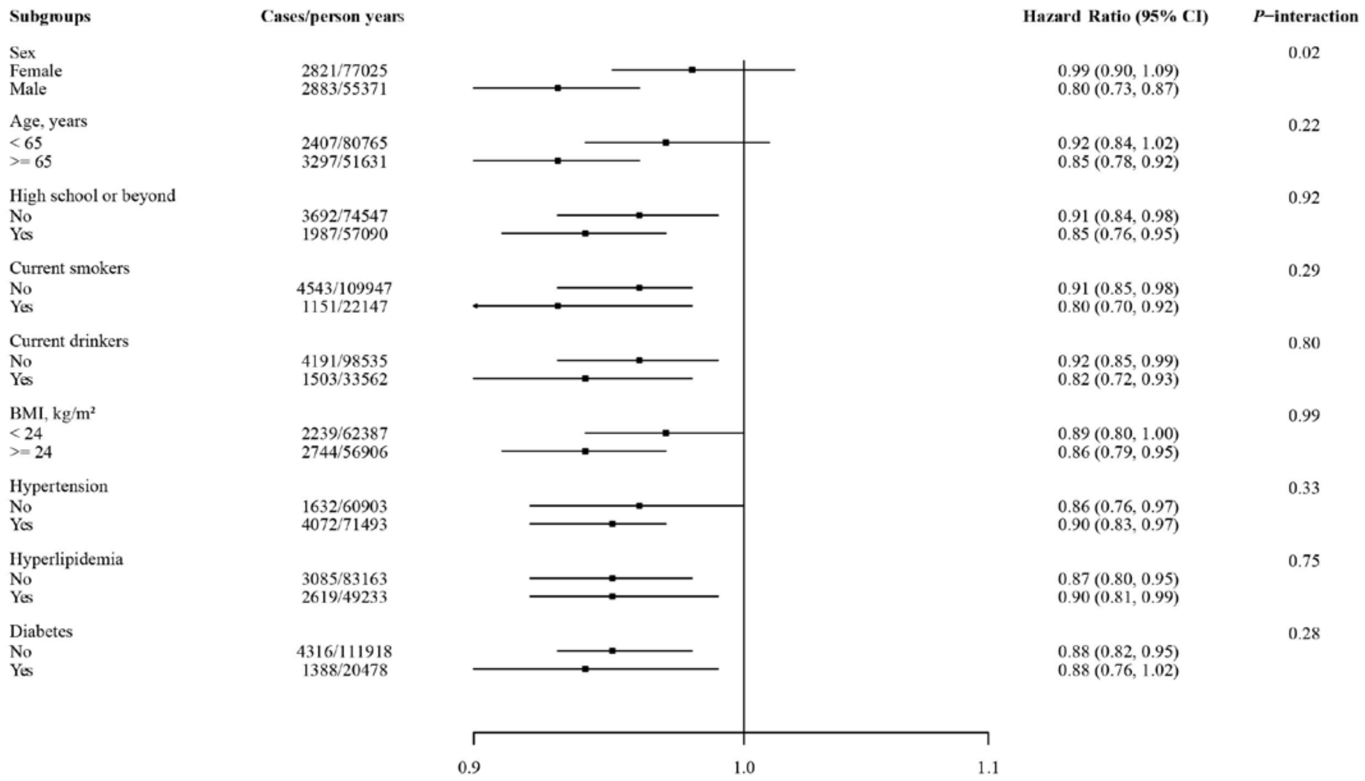

Supplementary Figure S1. Association of LTPA with incident CVD stratified by potential modifiers.

Hazard ratios were adjusted for age, sex, education, smoking status, alcohol intake, consumption of food (meat, vegetables, and fruit), hypertension, hyperlipidemia, diabetes, BMI, MET-hours/week, total sedentary time, and family history of CVD.

Hazard ratio is for every one increment in log10 [MET-hours/week] with incident CVD.

The number was 26435, 26526, 26523 and 23770 for education, smoking status, drinking status and BMI data, respectively.

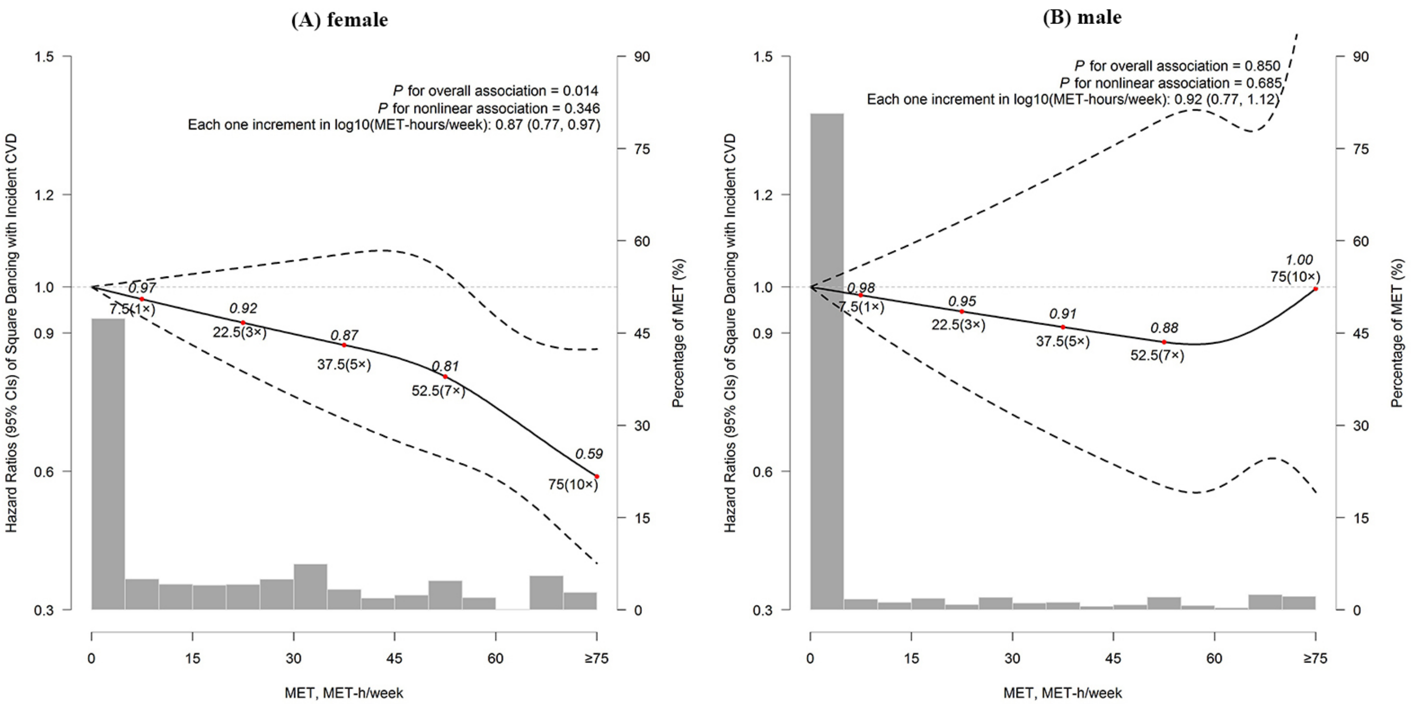

Supplementary Figure S2. The restricted cubic spline for the association of square dancing with incident CVD stratified by gender.

(A). Association between leisure-time physical activity and incident CVD in female.

(B). Association between leisure-time physical activity and incident CVD in male.

The multivariable models were adjusted for age, education, smoking status, alcohol intake, consumption of food (meat, vegetables, and fruit), hypertension, hyperlipidemia, diabetes, BMI, total sedentary time, other LTPA, and family history of CVD.

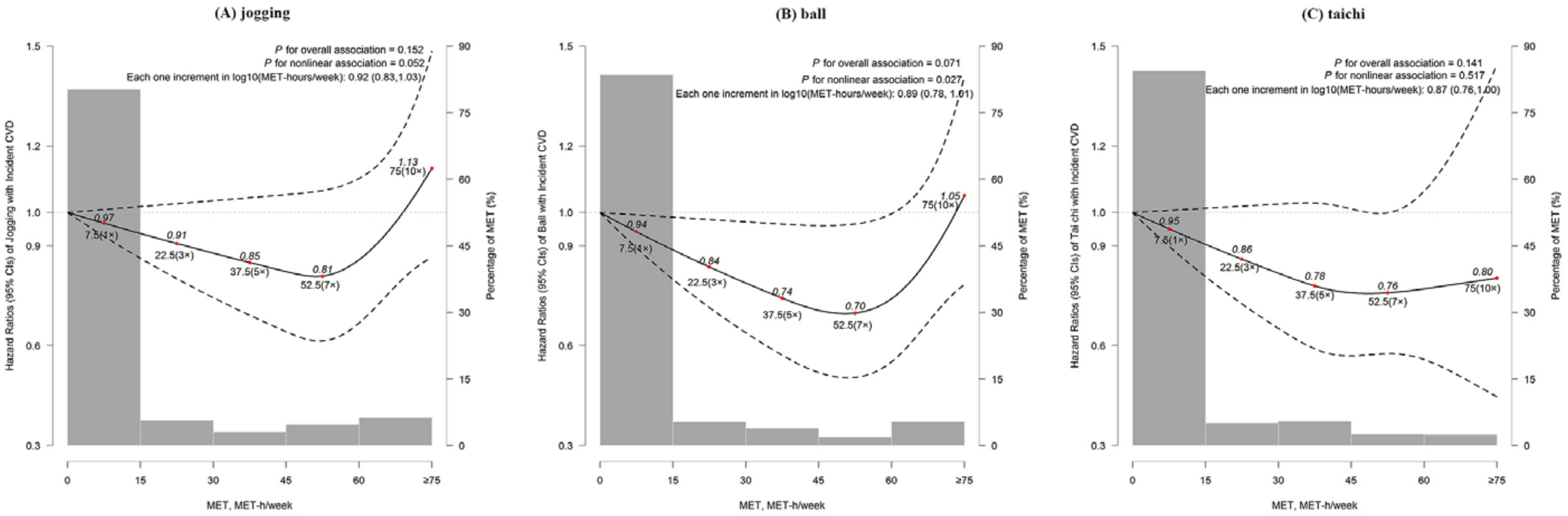

Supplementary Figure S3. Adjusted hazard ratios for CVD according to levels of different types of leisure-time physical activity.

(A). Association between jogging and incident CVD.

(B). Association between ball and incident CVD.

(C). Association between taichi and incident CVD.

The multivariable models were adjusted for age, sex, education, smoking status, alcohol intake, consumption of food (meat, vegetables, and fruit), hypertension, hyperlipidemia, diabetes, BMI, total sedentary time, other LTPA, and family history of CVD.

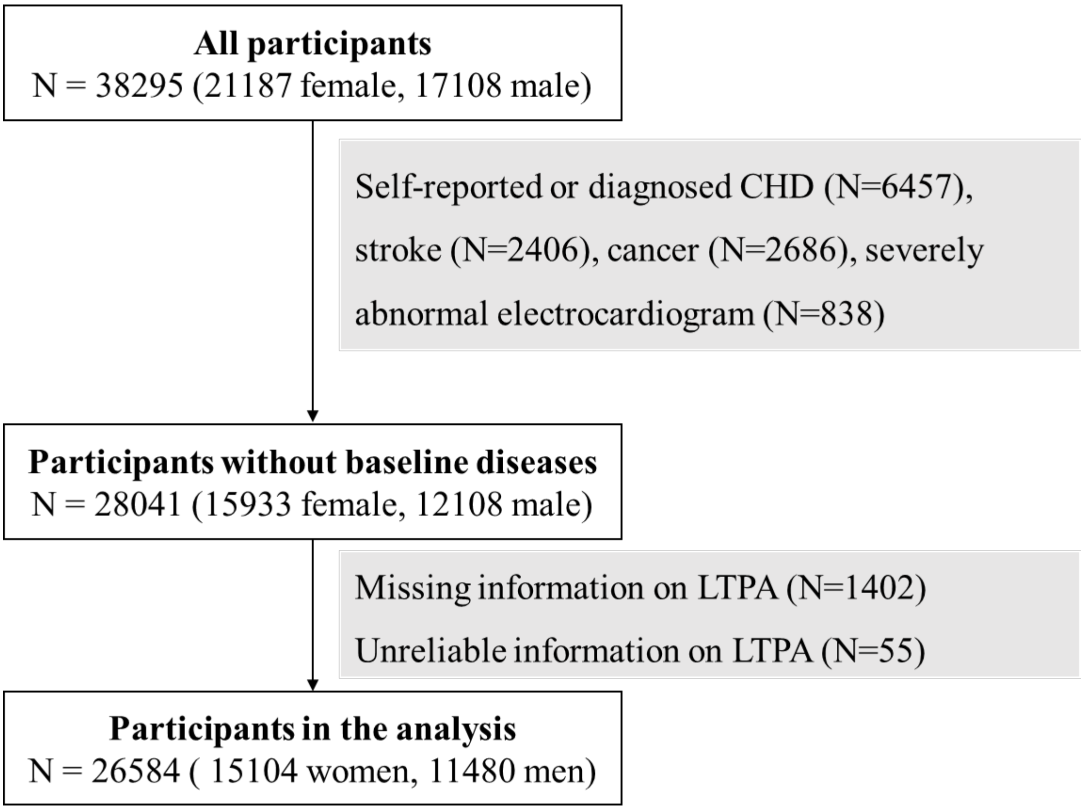

Supplementary Figure S4. The flowchart of study participants.

eReference

1. Ainsworth, B. E. *et al.* 2011 Compendium of Physical Activities: a second update of codes and MET values. *Medicine and science in sports and exercise* **43**, 1575-1581, doi:10.1249/MSS.0b013e31821ece12 (2011).
2. Chen, M. *et al.* Different physical activity subtypes and risk of metabolic syndrome in middle-aged and older Chinese people. *PloS one* **8**, e53258, doi:10.1371/journal.pone.0053258 (2013).
3. Tian, J. *et al.* Association of resting heart rate and its change with incident cardiovascular events in the middle-aged and older chinese. *Scientific reports* **9**, 6556 (2019).
